# Periodontal pathogens *Porphyromonas gingivalis* and *Fusobacterium nucleatum* promote tumor progression in an oral-specific chemical carcinogenesis model

**DOI:** 10.18632/oncotarget.4209

**Published:** 2015-06-08

**Authors:** Adi Binder Gallimidi, Stuart Fischman, Brurya Revach, Raanan Bulvik, Alina Maliutina, Ariel M. Rubinstein, Gabriel Nussbaum, Michael Elkin

**Affiliations:** ^1^ Sharett Oncology Institute, Hadassah-Hebrew University Medical Center, Jerusalem, Israel; ^2^ Institute of Dental Sciences, Hebrew University-Hadassah Faculty of Dental Medicine, Jerusalem, Israel

**Keywords:** oral cancer, TLR2, STAT3, IL-6, periodontitis

## Abstract

Oral squamous cell carcinoma (OSCC) is a lethal disease whose incidence is increasing. Epidemiologic studies demonstrate an association between periodontitis and oral cancer, and periodontal pathogens are implicated in the pathogenesis of numerous disorders, including rheumatoid arthritis, cardiovascular diseases, diabetes and gastrointestinal malignancies. Nevertheless, a causal role for periodontal pathogens in OSCC has not been shown, partly due to the lack of an appropriate animal model. Here, utilizing a newly-established murine model of periodontitis-associated oral tumorigenesis, we report that chronic bacterial infection promotes OSCC, and that augmented signaling along the IL-6-STAT3 axis underlies this effect. Our results indicate that periodontal pathogens *P. gingivalis* and *F. nucleatum* stimulate tumorigenesis via direct interaction with oral epithelial cells through Toll-like receptors. Furthermore, oral pathogens stimulate human OSCC proliferation and induce expression of key molecules implicated in tumorigenesis. To the best of our knowledge, these findings represent the first demonstration of a mechanistic role for oral bacteria in chemically induced OSCC tumorigenesis. These results are highly relevant for the design of effective prevention and treatment strategies for OSCC.

## INTRODUCTION

Chronic infections are increasingly recognized as an important epidemiologic/environmental determinant in cancer development. Almost 20% of human malignancies can be linked to infectious agents [1]. Among infection-related neoplasms, cancers of the stomach, liver and the uterine cervix are best characterized and attributed to *Helicobacter pylori*, hepatitis B and C viruses, and human papilloma virus, respectively. In addition to the role in initiation of several tumor types, chronic infection is believed to contribute to cancer progression by activating tumor-promoting signaling pathways (e.g., NF-kB, STAT3), thereby augmenting production of anti-apoptotic proteins, growth factors, and cytokines that foster cancer growth, dissemination and resistance to therapy [1–6].

Oral cavity squamous cell carcinoma (SCC) ranks among the top 10 most common cancers worldwide [7, 8]. The tongue is the most common anatomic site (up to 50%) of oral cancer in the western world [9]. The oral cavity, [including the tongue [10, 11], is naturally inhabited by microbial communities that exist in a balanced immunoinflammatory state with the host. However, certain species, such as *Porphyromonas gingivalis* (*P. gingivalis*), can disrupt this equilibrium, resulting in a dysbiotic host-microbiota interaction [6, 12]. Subsequently, other microbial species, such as *Fusobacterium nucleatum (F. nucleatum*), can become opportunistically pathogenic, and the combined effect of a dysbiotic microbial community along with a dysregulated immune response ultimately causes periodontal disease [6, 12], a chronic infectious/inflammatory condition that leads to soft tissue damage and eventually to bone resorption. Ironically, while involvement of these well-studied periodontal microorganisms in several types of gastrointestinal tract cancers (i.e., colon [13, 14], and pancreatic [15, 16]) was recently demonstrated, a mechanistic link between these bacteria and oral cavity SCC is much less established (despite the wealth of epidemiological data suggesting such involvement [5, 7, 17–20]).

Importantly, bacterial pathogens are often present in oral tumors, and chronic immune cell infiltration is an accompanying histological feature of oral cavity SCC progression [6, 7, 21, 22]. Nevertheless, a causal role of these pathogens in the etiology and progression of oral cavity SCC remains underappreciated [5], and further research is handicapped by the lack of an appropriate animal model.

Here we report the establishment of a new murine model of chronic infection-associated oral tumorigenesis, combining experimental mouse periodontitis [23] induced by *P. gingivalis* and *F. nucleatum*, along with administration of a specific oral carcinogen 4-nitroquinoline-1-oxide (4NQO) [24]. The choice of the bacteria was based on the fact that these pathogens are often present in the oral cavity and in tumor tissue [6, 7, 21, 22]. Furthermore, co-infection with *P. gingivalis* and *F. nucleatum* induces greater inflammation and bone resorption as compared to infection with each organism alone [23]. Utilizing this experimental system we found that chronic *P. gingivalis/F. nucleatum* infection profoundly affects oral cavity SCC progression and that augmented signaling along the IL-6-STAT3 axis may underlie this effect. Moreover, our results indicate that periodontal pathogens may stimulate tumorigenesis via direct interaction with cancerous and pre-cancerous oral epithelial cells, through activation of epithelial Toll-like receptors (TLR). Validating this mode of action in human oral cavity SCC cells *in vitro*, we detected that specific stimulators of TLR2/1 and TLR4 (which are expressed by SCC cells), or challenge with *P. gingivalis* and *F. nucleatum*, induce key molecular players mechanistically involved in oral tumor growth and aggressiveness (i.e., IL-6, cyclin D1, TNFα, MMP9, heparanase) [2, 7, 21, 25– 30]. Causal involvement of epithelial TLR signaling was further confirmed by applying TLR2 neutralizing antibody. Collectively, our findings provide experimental evidence that a periodontal pathogen-rich environment profoundly affects tumor progression in the oral cavity, conferring an aggressive phenotype to oral cavity SCC.

## RESULTS

### Mouse model of periodontal infection-associated oral tumorigenesis

**Figure 1:**
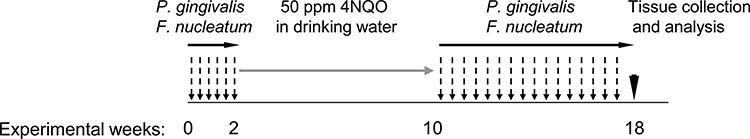
Schematic representation of the periodontal pathogen-associated oral tumorigenesis model Mice were administered with oral carcinogen 4NQO (50 ppm) in the drinking water for 8 weeks (grey arrow). In some of the 4NQO-treated mice chronic periodontitis was induced by repeated oral infection with a mixture of *P. gingivalis* and *F. nucleatum* every other day, initiated 2 weeks prior to 4NQO administration, and continued (2 times/week) until week 18 (black arrows). The non-infected mice were treated with vehicle alone. At the end of experimental week 18 mice were sacrificed (black arrowhead), their tongues harvested and processed for histological examination and immunostaining.

To elucidate the role of chronic infection in oral cavity SCC pathogenesis, we established and characterized a new experimental system (Figure 1) that combines the mouse model of chronic periodontitis [23] with the carcinogen 4-nitroquinoline-1-oxide (4NQO)-induced oral carcinoma model [24, 31]. Briefly, mice were administered 4NQO for 8 weeks, as described in [24, 32, 33] and further detailed in Methods (Figure 1, grey arrow). A group of the 4NQO-treated mice were repeatedly infected with a mixture of two major human periodontal pathogens, *P. gingivalis* and *F. nucleatum* suspended in CMC, as described in Methods (Figure 1, dashed arrows). Infection with both bacteria is a reliable model of experimental periodontitis, known to induce a pronounced inflammatory response in the gingiva that leads to osteoclast activation and alveolar bone resoption [23]. The non-infected 4NQO-treated mice were administered CMC alone. Of note, both bacteria were recovered from the tongue surface following oral challenge by swabbing and culturing, and by PCR (Supplementary Figure 2A), and chronic challenge led to an infiltrate dominated by macrophages in the tongue sub-epithelium (Supplementary Figure 2B). Thus, in addition to aggregating within biofilms on the tooth surface and within the gingival crevice, in this experimental model *P. gingivalis* and *F. nucleatum* are present on the mouse tongue surface. These observations are in agreement with findings in human oral cavity, where periodontal bacteria are abundantly present on the tongue dorsum [11] with similar prevalence as in the subgingival biofilm [10, 34].

4NQO was administered for 8 weeks (as in [24, 32, 33]) and since we hypothesized that infection may enhance tumorigenesis, the mice were sacrificed on week 18 (Figure 1), a relatively early time point compared to other studies [31]. Tongues were excised and serial H&E stained sections were evaluated in a blinded fashion by an oral pathologist (S. F.). Tongue carcinoma was observed in 6 out of 7 infected mice and in 5 out of 7 non-infected mice. All carcinomas were graded as moderately differentiated except for one in the group infected with bacteria that was graded as poorly differentiated.

**Figure 2:**
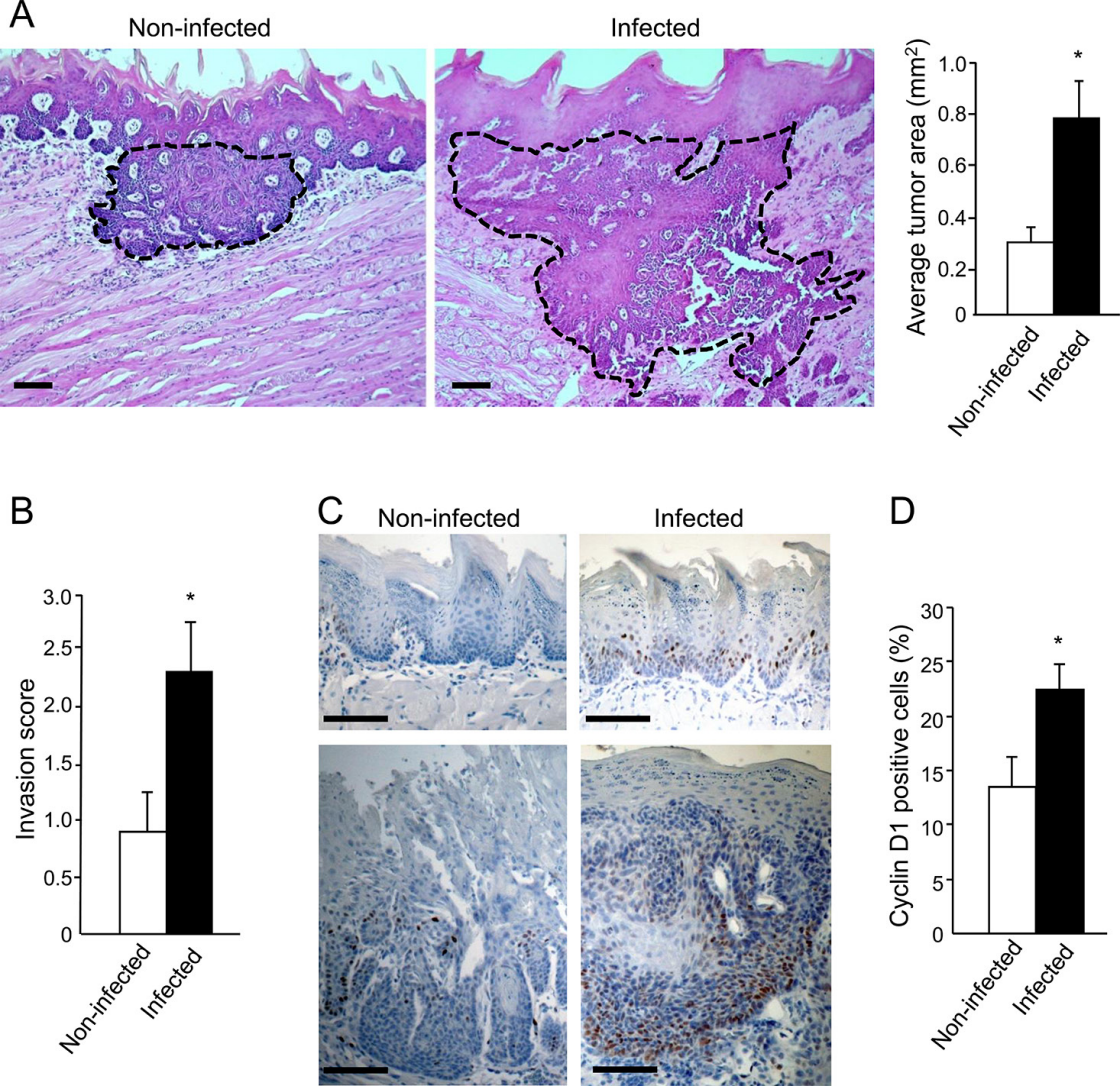
Increased tumor severity in *P. gingivalis/F. nucleatum* infected mice **A, B.** Serial tongue sections (5 μm thick) harvested from 4NQO-treated, non-infected and infected mice on week 18 were stained with H&E and visualized with a Zeiss axioscope microscope. A. The area of each neoplastic lesion was determined with Axio Vision Image software. Left, middle. Representative microphotographs of H&E stained tongue sections. Tumors are delineated by dashed lines; Scale bars: 200 μm. Right. Average tumor area in non-infected (empty bar) and infected (black bar) mice on experimental week 18. Data are the mean ± SE, *n* = 5 mice per condition; *p = 0.004 (Mann-Whitney). B. Invasive score of the tumors found in non-infected (empty bar) and infected (black bar) mice was determined as described in Methods and in Supplementary Figure 1. Data are the mean ± SE, *n* = 7 mice per condition, *p = 0.029 (Mann-Whitney). **C, D.** Immunostaining with anti cyclin D1 antibody reveals enhanced expression of cyclin D1 in non-cancerous (top) and cancerous (bottom) tongue epithelium of infected vs. non-infected mice. C. Representative images of cyclin D1 immunostained sections are shown; Scale bars: 100 μm. D. Percentage of Cyclin D1-immunostained cells in the epithelium was quantified as the number of positively stained cells per existing total epithelial cells, based on [58] in 5 randomly selected high-power microscopic fields per mouse at magnification × 200. Data are the mean ± SE, *n* = 4 mice per condition *p = 0.0026 (Student's *t*-test).

Morphometric and immunohistochemical analysis revealed that *P. gingivalis*/*F. nucleatum* chronic infection markedly enhanced the severity of the tongue tumors. Tumors from infected mice, in comparison to non-infected mice, were 2.5 times larger (*p* < 0.05, Figure 2A), and were significantly more invasive (Figure 2B). Furthermore, the expression of cyclin D1, a pivotal oncogene in experimental [25] and human [26] oral tumorigenesis, was significantly enhanced in infected vs. non-infected mice, both in cancerous and non-cancerous tongue epithelium (Figure 2C, 2D).

**Figure 3:**
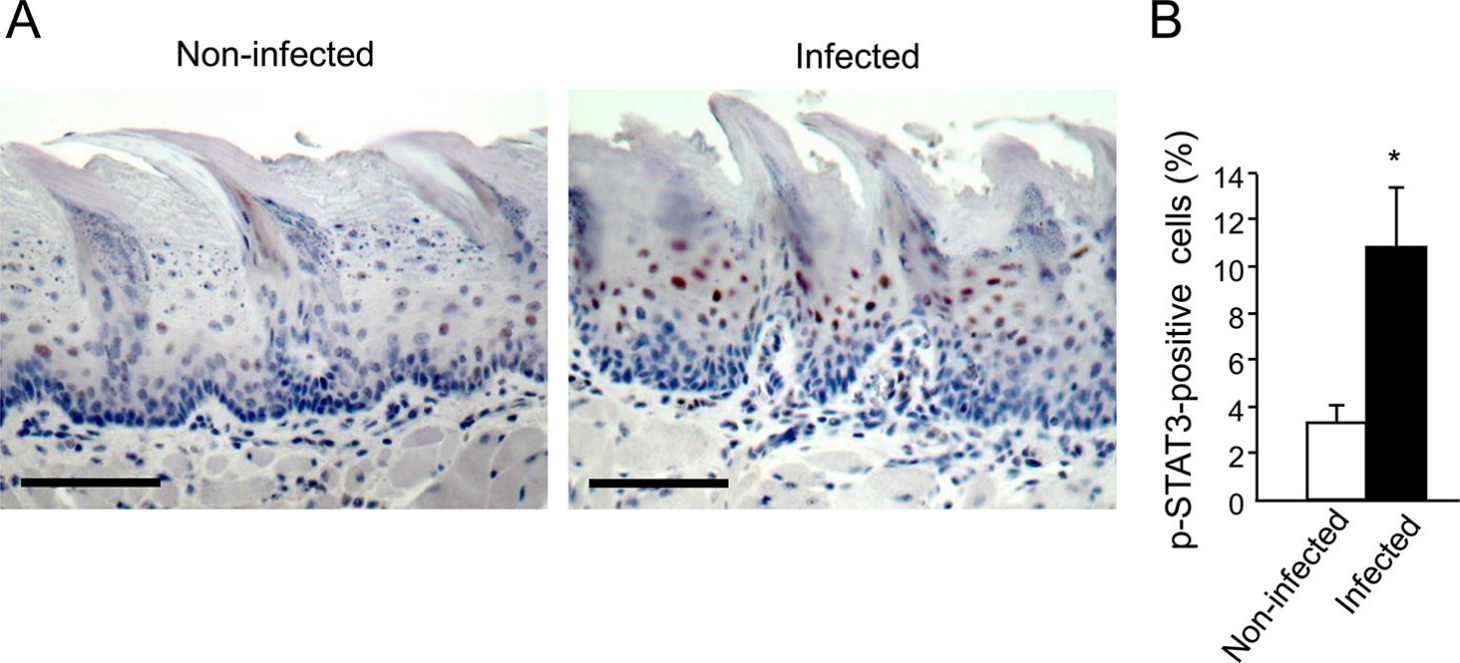
Increased STAT3 activation in tongue epithelium of infected mice C57/Bl6 mice were infected with a mixture of *P. gingivalis* and *F. nucleatum* (as described in Methods) every other day for 6 days. Mice were sacrificed twenty-four hours after the last infection, their tongues removed and processed for immunostaining with anti-pSTAT3 antibody. **A.** Immunostaining with anti pSTAT3 antibody reveals increased levels of nuclear-localized pSTAT3 in tongue epithelium of infected (right) vs. non-infected (left) mice. Representative images of are shown. Magnification × 200 **B.** Percentage of pSTAT3-immunostained cells in the epithelium was calculated in ≥5 microscopic fields per mouse. Data are the mean ± SE, *p = 0.01 (Student's *t*-test). Scale bars: 100 μm.

We next hypothesized that in infected mice the periodontal pathogens, rather than 4NQO, may be mainly responsible for the upregulation of cyclin D1, acting via a STAT3 dependent-mechanism. Indeed, STAT3 is an important mediator of oral cavity SCC tumorigenesis in clinical and experimental settings [35–39]. STAT3 is one of the key signaling molecules which is responsible for induction of cyclin D1[40], although additional pathways (e.g. MAPK-ERK, Wnt) are known to regulate cyclin D1 as well. STAT3 also controls additional genes driving proliferation, suppression of apoptosis and aggressive tumor behavior [40]. Interestingly, the STAT3 pathway is reportedly activated in oral epithelial cells cultured *in vitro* in the presence of *P. gingivalis* [41, 42]. To test this hypothesis, we analyzed the activation status of STAT3 in tongue epithelium of mice infected with periodontal pathogens but not exposed to 4NQO. Immunostaining for phosphoSTAT3 (pSTAT3) revealed that three consecutive administrations of *P. gingivalis*/*F. nucleatum* sufficed to markedly induce STAT3 activation in the tongue epithelium, as evidenced by a more than 3-fold increase (*p* = 0.01) in the number of cells positive for nuclear-localized pSTAT3 in infected vs. non-infected mice (Figure 3). These findings are in agreement with the proposed role of STAT3 in fostering oral tumorigenesis [35–39], and may explain elevated levels of the STAT3 target gene cyclin D1 found in both cancerous and non-cancerous tongue epithelium of *P. gingivalis*/*F. nucleatum* -infected mice (Figure 2C, 2D).

**Figure 4:**
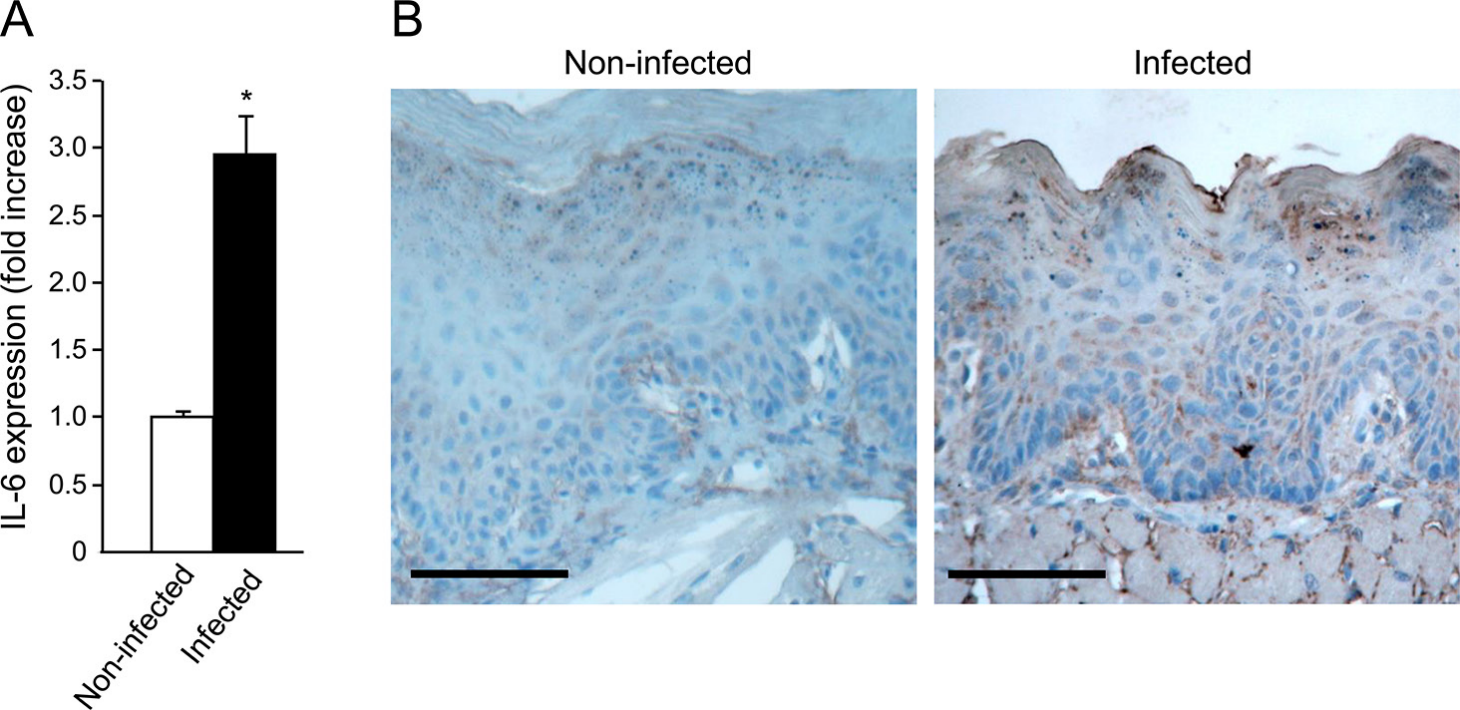
Increased IL-6 levels in tongue epithelium of infected mice **A**. Mice non-infected (white bar) or infected (black bar) with a mixture of *P. gingivalis* and *F. nucleatum* every other day for 6 days (as described in Methods) were sacrificed four hours after the last infection. The tongue mucosa was isolated and processed for RNA. Quantitative RT-PCR analysis revealed a 3 fold increase in IL-6 mRNA levels in the tongue mucosa of infected mice. Data are the mean ± SE, *p = 0.014 (Student's *t*-test). **B**. Immuno-histochemical analysis with anti-IL-6 antibody revealed expression of IL-6 protein in epithelial compartment of the tongue tissues harvested non-infected and infected mice treated with 4NQO as described in Methods and in Figure 1. Scale bars: 100 μm.

STAT3 activation is induced by several cytokines, most notably IL-6 [43]. We therefore studied the effect of *P. gingivalis*/*F. nucleatum* infection on IL-6 expression in mouse tongue tissue. As shown in Figure 4A, quantitative RT-PCR analysis revealed a 3 fold increase in IL-6 mRNA levels in tongues harvested from mice following repeated infection with *P. gingivalis*/*F. nucleatum*. Given the increase in macrophage infiltration found in the tongues of *P. gingivalis*/*F. nucleatum* infected mice (Supplementary Figure 2B), and the known role of macrophages in IL-6 production, it would be straightforward to assume that the observed increase in IL-6 expression (Figure 4A) occurs primarily in macrophages. However, double-immunofluorescent staining with antibodies directed against F4/80 and IL-6 failed to reveal co-localization of IL-6 with macrophages in the tongues of infected mice. Moreover, immunochemical analysis revealed that epithelial cells *per se* represent a key source of IL-6 in this experimental system (Figure 4B).

### *In vitro* effects of *P. gingivalis* and *F. nucleatum* on human oral cavity SCC cells

**Figure 5:**
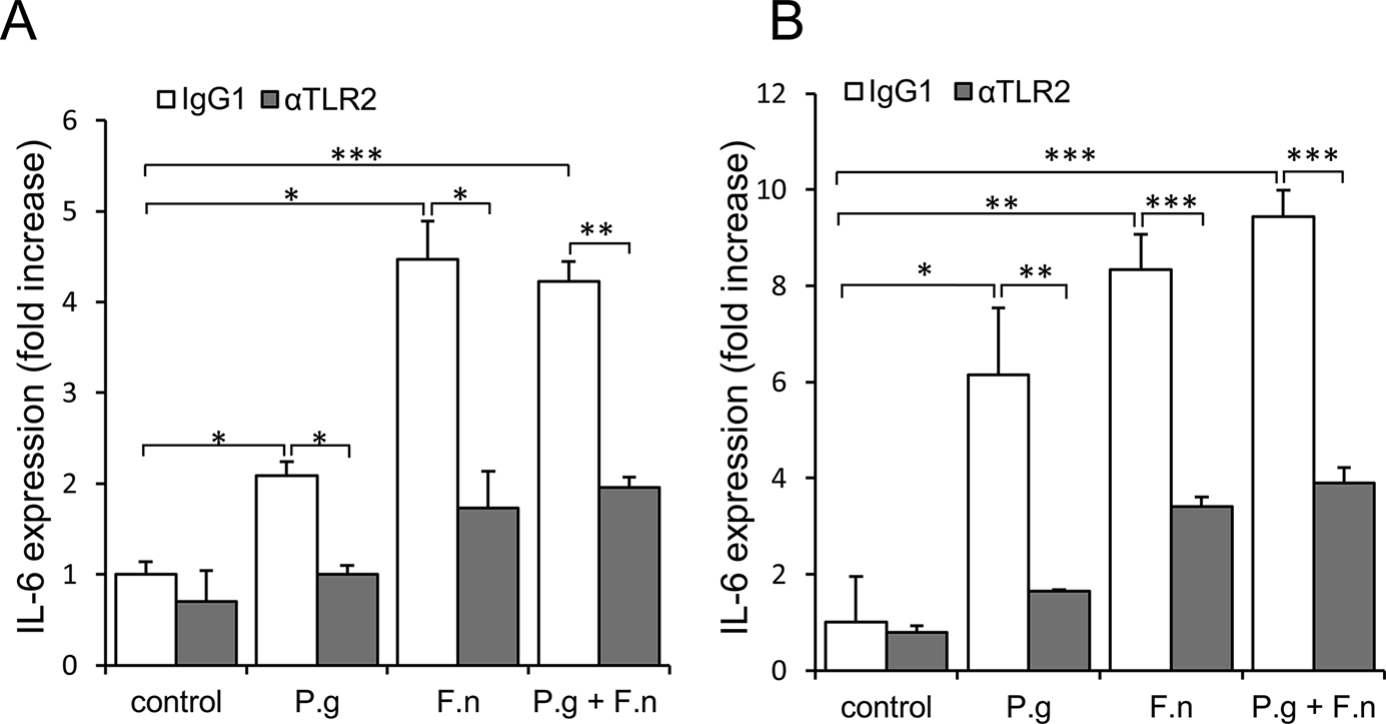
Increased expression of IL-6 in oral cavity SCC cell lines following co-culture with P. *gingivalis* and F. *nucleatum* SCC-25 **A.** and CAL 27 **B.** cells were cultured alone (control) or with *P*. *gingivalis* (P.g), *F*. *nucleatum* (F.n), or mixture of P. *gingivalis* and F. *nucleatum* (P.g + F.n), as described in Methods. In some plates neutralizing antibody directed against TLR2 (αTLR2) was added to SCC-25 (A, filled bars) and CAL 27 (B, filled bars) cells, cultured alone (control) or with P.g, F.n, or mixture of P.g + F.n as described in *Methods*. Empty bars: isotype control (IgG1). Quantitative RT-PCR was used to assess expression of IL-6. Data are the mean ± SE; *p < 0.05, **p < 0.01, ***p < 0.001.

The above findings, together with reports that epithelial cells of the oral cavity express TLRs [22, 44, 45], led us to speculate that *P. gingivalis* and *F. nucleatum* trigger TLR signaling in pre-cancerous/cancerous epithelium, resulting in overexpression of epithelial-derived IL-6. To validate the proposed mode of action, we co-incubated *P*. *gingivalis*/*F*. *nucleatum* with tongue epithelium-derived oral cavity SCC cell lines SCC-25 and CAL 27. We first verified the expression and functionality of TLR2 and TLR4 in SCC-25 and CAL 27 cells by RT-PCR and by production of IL-6 in response to treatment with ligands specific for TLR2/1 (lipopeptide PAM3CSK4) or TLR4 (lipopolysaccharide, LPS) (Supplementary Figure 3). Moreover, as shown in figure 5, and in agreement with *in vivo* observations (Figure 4), exposure to each of the pathogens (or to a mixture of both) significantly increased the expression of IL-6 by SCC-25 (Figure 5A, empty bars) and CAL27 (Figure 5B, empty bars) cell lines. Importantly, TLR2 inhibition markedly inhibited pathogen-induced IL-6 expression (Figure 5, filled bars), whereas treatment with a TLR4 inhibitor did not interfere with pathogen-induced IL-6 expression, consistent with the dominant role of TLR2 in the innate response to *P. gingivalis* and *F. nucleatum* demonstrated by us and others [46–50].

The *in vitro* findings demonstrating involvement of TLR2 (Figure 5) are in agreement with the observation that *in vivo* infection with *P. gingivalis*/*F. nucleatum* induced NF-κB signaling (a known consequence of TLR activation [45]) in mouse tongue epithelium, as evidenced by a 3-fold increase in the percentage of epithelial cells positive for nuclear p65 in infected vs. non-infected mice 6 days post infection (24 ± 2.6% vs. 7% ± 1.5, *P* < 0.01). Of note, exposure of oral cavity SCC cells to the periodontal pathogens resulted in the induction of additional cytokines, enzymes and bioactive molecules implicated in oral cavity SCC proliferation, survival and aggressiveness (i.e., cyclin D1, TNFα, MMP9, and heparanase [21, 25–30]) (Supplementary Figure 4).

**Figure 6:**
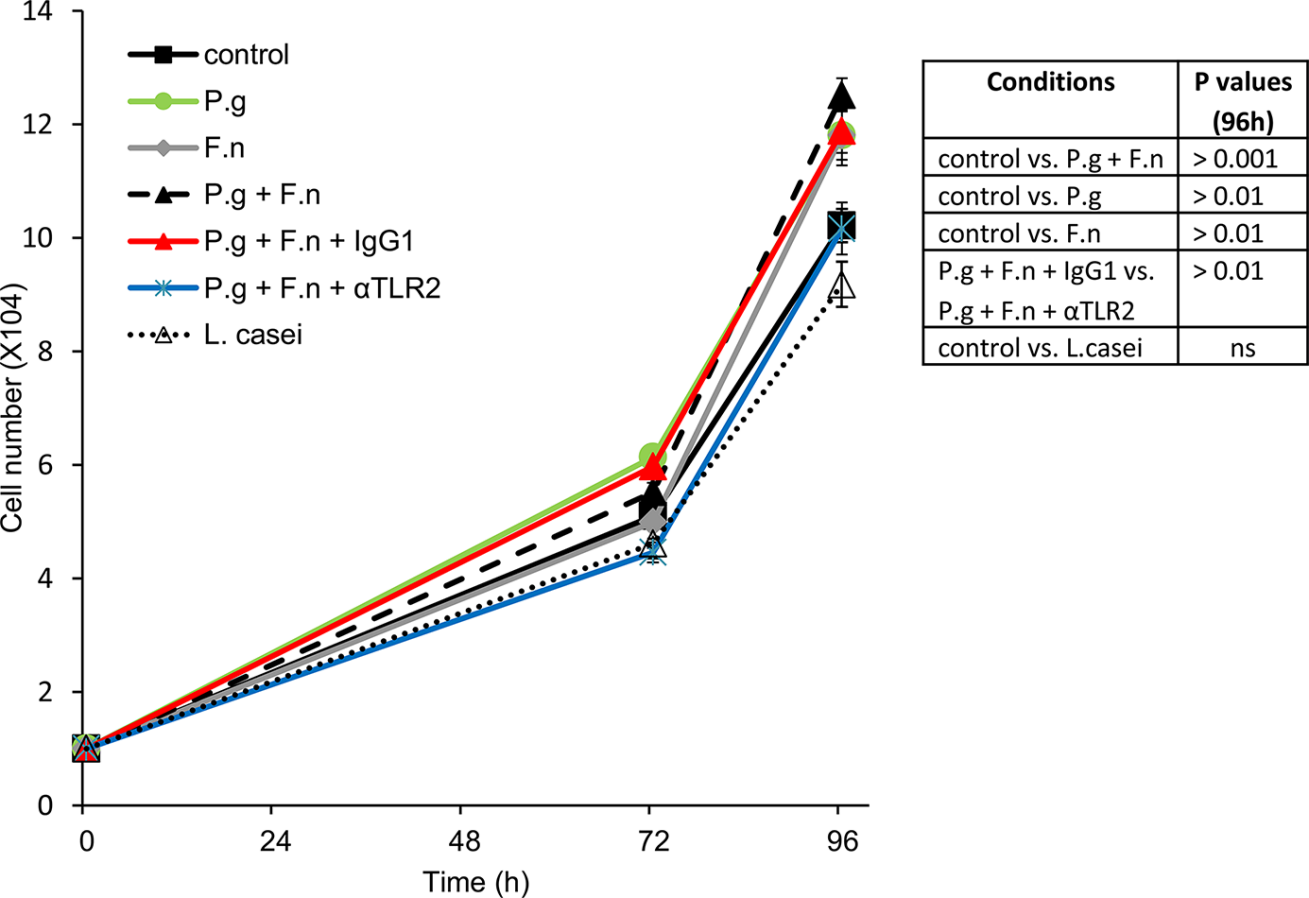
*P. gingivalis* and *F. nucleatum* stimulate *in vitro* proliferation of human SCC cells SCC-25 cells were plated on 24-well plates at 10^4^ cells per well (in quadruplicates) and cultured either alone (control) or in the presence of *P*. *gingivalis* (P.g), *F*. *nucleatum* (F.n), mixture of both (P.g + F.n), or *L*. *Casei*, as described in *Methods*. In some plates αTLR2 or the isotype control (IgG1) was added one hour prior to mixture of P.g + F.n. Cell numbers were determined as described in *Methods*. Data are the mean ± SE. **Inset**: *p* values determined by Student's *t*-test.

Finally, co-incubation with either *P*. *gingivalis*, *F*. *nucleatum* or a mixture of both significantly stimulated *in vitro* proliferation of human oral cavity SCC cells (Figure 6), in further agreement with the augmented tumor growth observed under *P. gingivalis*/*F. nucleatum* infection *in vivo* (Figure 2). Moreover, inhibition of TLR2 with neutralizing antibody abrogated the effect of *P. gingivalis*/*F. nucleatum* on SCC-25 proliferation, supporting the role of TLR2 in the pro-tumorigenic effects of the pathogens (Figure 6)

Of note, oral cavity SCC cell proliferation was not affected by co-incubation with *L. casei* (commensal bacteria found in the human intestine and mouth, which is not implicated in carcinogenesis) (Figure 6), suggesting that the effects of periodontal pathogens in this setting cannot be generalized to all oral bacteria.

## DISCUSSION

*P*. *gingivalis* and *F*. *nucleatum*, best known for their involvement in periodontitis, have been implicated in the pathogenesis of several chronic diseases, as well as various types of gastrointestinal malignancies (e.g. colorectal, pancreatic) [13–16]. Paradoxically, an involvement of these oral pathogens in the etiology and progression of oral cavity SCC is much less established, despite the wealth of epidemiological data suggesting such involvement [5, 6, 19]. To address the paucity of mechanistic approaches for testing the effects of periodontal bacteria in oral cavity SCC, we established an animal model of chronic P. *gingivalis*/F. *nucleatum* infection-associated oral tumorigenesis. Here we demonstrate that *P. gingivalis*/*F. nucleatum* chronic infection promotes the growth and severity of 4NQO-induced tongue tumors (Figure 2); we further show that augmented signaling along the IL-6-STAT3 axis likely underlies this effect (Figures 3–5).

Similar to other anatomic sites, chronic infection in the oral cavity is likely to contribute to tumorigenesis by several mechanisms, including aberrant activation of infiltrating immune cells, induction of DNA damage through generation of reactive oxygen and nitrogen species and increased levels of immunocyte-derived bioactive molecules that facilitate tumor progression [7, 43]. In addition to these mechanisms, driven mainly by activated immunocytes, the novelty of our findings lies in the demonstration that periodontal pathogens directly stimulate cancerous cells of epithelial origin, resulting in induction of SCC-promoting factors (Figure 5 and Supplementary Figure 4) and augmented cell growth (Figure 6). Moreover, applying a neutralizing antibody approach, we show an important role for epithelial-expressed TLR2 in this process. These findings are consistent with the recently demonstrated role of epithelial TLR2 in the progression of non-oral carcinomas (i.e. intestinal and breast) [51]. Indeed, due to the pivotal role of TLRs in immune responses, the majority of studies aimed at understanding TLR biology have focused on immunocytes, rather than on tumor cells. However, TLRs appear to be expressed by tumor cells as well [44, 45, 52], including those originating from the normal oral mucosa and oral cancer (Ref. [22] and present study). These and other reports [15, 46, 47, 53], together with our findings that oral cavity SCC cells respond to *P*. *gingivalis*/*F. nucleatum* in a TLR2-dependent manner, (Figures. 5, 6, and Supplementry Figures 3, 4), strengthen the notion that triggering of epithelial TLRs facilitates tumorigenesis in the oral cavity, although the involvement of additional receptors/pathways cannot be excluded [54]. Consistent with the TLR involvement, infection with *P. gingivalis*/*F. nucleatum in vivo* induced NF-κB signaling in mouse tongue epithelium, manifested by a higher number of epithelial cells positive for nuclear p65 NF-κB in infected vs. non-infected mice (24% vs. 7%, *P* < 0.01). Moreover, *in vitro* induction of epithelial IL-6 and TNFα (well-known TLR-controlled, NF-κB-inducible cytokines [55]) by periodontal pathogens (Figure 5 and Supplementry Figure 4, left), is also in agreement with activation of a TLR-2-NF-κB signaling pathway. Thus, in the evolving story of microbial/mucosal/immune interplay in the pathogenesis of oral cavity SCC, our study adds a new level of complexity, highlighting the role of a direct interaction between pathogens and epithelial cells. Collectively, our results suggest that exposure of oral epithelial cells to *P. gingivalis/F. nucleatum* triggers TLR signaling, resulting in IL-6 production that activates STAT3 which in turn induces important effectors driving oral cavity SCC growth and invasiveness (i.e., cyclin D1, MMP9, heparanase). Although further studies are warranted to fully unravel the intricate network of molecular and cellular events underlying the action of periodontal pathogens in oral cavity SCC tumorigenesis (including the contribution of extracellular bacteria and their products vs. intracellular bacteria), our present findings underscore the importance of epithelial-bacterial interactions in this complex network. Our model may offer new approaches not only to the further understanding of this connection, but also to the design of novel prevention/treatment strategies for oral cavity SCC in the setting of chronic oral infection.

## MATERIALS AND METHODS

### *In vivo* carcinogenesis model

Ten- to twelve-week-old Balb/c male mice were obtained from Harlan Laboratories (Jerusalem, Israel), housed at 2–5 per cage, provided with water and regular chow diet *ad libitum*, and maintained in a 12-h/12-h light/dark cycle. Mice were randomly divided into two groups (*n* = 7 mice per group) and administered 50 ppm 4NQO for 8 weeks in their drinking water. One group was repeatedly infected with a mixture of *P. gingivalis* (strain 381) and *F. nucleatum* (strain PK1594) (300 μl of 10^10^ bacteria/ml and 100 μl of 10^9^ bacteria/ml respectively) suspended in 2% carboxymethylcellulose (CMC), as described previously [23]. Mice in this group were orally infected 3 times per week starting two weeks prior to 4NQO administration. Infection was repeated at 2 times per week for 8 weeks after 4NQO withdrawal, as shown in Figure 1. During the period of 4NQO administration mice were not infected with the bacteria mixture (4NQO has been previously reported to exert anti-bacterial action [56]). The second group was treated with vehicle (CMC) alone. All mice were sacrificed on week 18, and their tongues were harvested and processed for histological examination and immunostaining. Animal experiments were approved by the Institutional Animal Care and use Committee.

### Tumor histopathology and immunostaining

Tongues were fixed in 4% paraformaldehyde, embedded in paraffin, and thin-sectioned (5 μm). Every 20th section of the tissues was stained with H&E and visualized with a Zeiss axioscope microscope. Neoplastic lesions were scored in a blinded fashion by an expert oral pathologist (S.F.). The size of each neoplastic lesion was determined with Zeiss Image software (AxioVision) as previously described [57].

Tumor invasion was graded from 0 to 3 as follows (Supplementary Figure 1): 0 = no evidence of invasion, 1 = malignant cells deep to basal cell layer, no invasion into striated muscle; 2 = minor invasion into striated muscle; 3 = invasion through the full depth of striated muscle. For each mouse, the entire tongue was reviewed and all tumors were graded. Seven mice per experimental condition were analyzed, and the highest score obtained for each mouse was used to calculate the invasiveness index. Immunostaining of the paraffin-embedded sections was performed as previously [57]. Primary antibodies used were anti-cyclin D1 (1:50, Thermo SCIENTIFIC, #RM9104-S0), anti-p-Stat3 (1:100, Cell Signaling, D3a7), anti-NF-κB p65 (1:400, Cell signaling, D14E12), anti-F4/80 (1:400, Serotec, MCA497GA) and anti-IL-6 (1:400, Abcam, ab6672). Color was developed using the DAB substrate kit (Thermo Scientific). F4/80 positive cells were quantified as cells per mm epithelial length, including all epithelial layers and stroma above the muscle layer, as described [58]. Nuclear p-STAT3, cyclin D1 and p65 NF-kB -positive cells were quantified as the number of positively stained cells per existing total epithelial cells [58].

### Bacterial and eukaryotic cell culture

*P. gingivalis* and *F. nucleatum* were grown in Wilkins media (Oxoid, Basingstoke, UK) for 2 or 1 days respectively, under anaerobic conditions at 37°C. *Lactobacillus casei* (*L. casei*) was grown in M.R.S broth media (Oxoid, Basingstoke, UK) for 1 day under aerobic conditions at 37°C. The bacteria were washed 3 times with sterile phosphate-buffered saline (PBS) before use. SCC-25 and CAL 27 human tongue SCC cell lines, originally obtained from ATCC, were generously provided by Dr. I. Vlodavsky (Technion, Haifa, Israel). CAL-27 cell line was isolated from the lesion taken prior to treatment from a 56 year old male [59]; SCC-25 line was isolated from a tongue of 70 year old male [60]. Both lines are extensively characterized, widely used in oral cavity SCC studies [61], HPV-negative, have relatively long doubling times (35 h) and are non-invasive *in vivo*, as proven by the xenograft models [60, 62]. Cells (tested negative for mycoplasma) were grown in DMEM supplemented with 1 mM glutamine, 50 μg/ml streptomycin, 50 U/ml penicillin, and 10% fetal calf serum (Biological Industries, Israel) at 37^°^C and 8% CO2.

### *In vivo* infection with P. gingivalis and F. nucleatum

Male Balb/c mice were orally infected every other day for 6 days with a mixture of *P. gingivalis and F. nucleatum* (300 μl of 10^10^ bacteria/ml and 100 μl of 10^9^ bacteria/ml respectively). Mouse tongues harvested at the time points specified in the text were processed for immunostaining or snap-frozen for RNA isolation. Three mice per condition/time point were analyzed.

### Cell co-culture with bacteria and RNA isolation

SCC-25 and CAL 27 were plated in 12-well plates at 2 × 10^5^ cells per well in growth medium overnight. The cell confluence at the time of infection was ≤80%. One hour prior to bacterial infection medium was changed to DMEM without streptomycin and penicillin and cells were incubated with *P. gingivalis* (MOI 100), *F. nucleatum* (MOI 5), a mixture of both, or left untreated. Specific anti-TLR2 neutralizing antibody (20 microg/ml, clone T2.5, eBioscience, Inc) or isotype control (clone MOPC 21) was added to some plates (as indicated in the text) one hour prior to addition of bacteria. Following 4 or 24 hours of incubation, cells were lysed and processed for RNA isolation using TRIzol (Invitrogen), according to the manufacturer's instructions.

### Analysis of gene expression by qRT-PCR

Total RNA was isolated from snap-frozen tissue samples or cultured cells using TRIzol (Invitrogen), according to the manufacturer's instructions, and quantified by spectrophotometry. After oligo (dT)-primed reverse transcription of 1 μg total RNA, the resulting single stranded cDNA was amplified using the primers listed below. Real-time quantitative PCR (qRT-PCR) analysis was performed with an automated rotor gene system RG-3000A (Corbett research, Sydney, Australia). The PCR reaction mix (20 μl) was composed of 10 μl QPCR sybr master mix (Finnzymes, Espoo, Finland), 5 μl of diluted cDNA (each sample in triplicate) and a final concentration of 0.3 μM of each primer. Hypoxanthine guanine phosphoribosyl transferase (HPRT) primers designed in the lab using Primer-BLAST software (NCBI) were used as an internal control [63]. The following primers were utilized:

Human HPRT F: 5′-GCTATAAATTCTTTGCTGA CCTGC-3′, R: 5′-ATTACTTTTATGTCCCCTGTTG ACTG-3′.

Human IL-6 F: 5′-GGCACTGGCCAGAAAAC AACC-3′,

R: 5′-GCAAGTCTCCTCATTGAATCC-3′.

Human MMP9 F: 5′-CCTGGAGACCTGAGAACC AATC-3′,

R: 5′-CCACCCGAGTGTAACCATAGC-3′.

Human TNFα F: 5-CTGCCCCAATCCCTTTATT-3′,

R: 5′-CCCAATTCTCTTTTTGAGCC-3′.

Human Cyclin D1 F: 5′-TGTTCGTGGCCTCTAA GATGAAG-3′,

R: 5′-AGGTTCCACTTGAGCTTGTTCAC-3′.

Human heparanase F: 5′-CCAGCACGGAAATGAA AGA-3′,

R: 5′-TCGATGGTGATGGACAGGAA-3′.

### Cell proliferation assay

Cells were plated in 24-well plates at 10^4^ cells per well in the growth medium. One hour prior to bacterial infection medium was changed to DMEM without streptomycin and penicillin and cells were incubated with *P. gingivalis* (MOI 10), *F. nucleatum* (MOI 1), a mixture of both *P. gingivalis* and *F. nucleatum*, *L*. *Casei* (MOI 10), or left untreated. Cell numbers were counted at indicated time points using a hemocytometer. Each experiment was performed in quadruplicates and repeated at least twice.

### Recovery of *P. gingivalis* and *F. nucleatum* from mouse tongues

A sterile cotton swab was rubbed against the tongue surface of infected and non-infected mice, 4 hours after infection for 20 seconds and then vortexed in 100 μl of prereduced transport media (Yeast Extract 5 g/L, Proteose Peptone 1 g/L, Cysteine-HCl 0.5 g/L, NaCl 8.5 8.5 g/L, Na2HPO4 0.868 g/L, KH2PO4 0.528 g/L, 0.1% Tween-80, 15% Glycerol, pH = 7). An aliquot, plated onto supplemented blood agar, was incubated anaerobically for 2 weeks. *P*. *gingivalis* and *F*. *nucleatum* colonies were identified by their black pigmentation and yellow-gray color (respectively) and by PCR with primers specific for *P. gingivalis* 16S rRNA and *F. nucleatum*. PCR conditions were as follows: an initial denaturation step at 95^°^C for 10 minutes, 30 cycles of denaturation at 94^°^C for 15 seconds, hybridization at 61^°^C for 30 seconds, and elongation at 72^°^C for 30 seconds. Primers used were:

*P*. *gingivalis* 16S rRNA F: 5′-CTTGACTTCAG TGGCGGCAG-3′

R: 5′-AGGGAAGACGGTTTTCACCA-3′

*F*. *nucleatum* F: 5′-CAACCATTACTTTAACTC TACCATGTTCA-3′

R: 5′-ATTGACTTTACTGAGGGAGATTATGTAA AAATC-3′.

### Statistics

Statistical differences were analyzed by the unpaired Student's *t* test or the Mann-Whitney test. *P* values < 0.05 were considered statistically significant. All statistical tests were two-sided.

## SUPPLEMENTARY FIGURES


