# Supplementary material for: Periodontal pathogens Porphyromonas gingivalis and Fusobacterium nucleatum promote tumor progression in an oral-specific chemical carcinogenesis model
Source: Oncotarget. 2015 Jun 8;6(26):22613–23. doi: 10.18632/oncotarget.4209 (PMC4673186; doi:10.18632/oncotarget.4209)
Supplement: Supplementary file 1 [file oncotarget-06-22613-s001.pdf]

SUPPLEMENTARY FIGURES

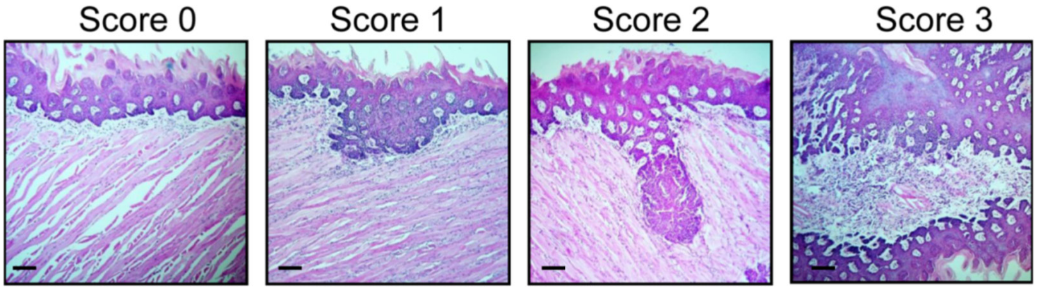

Supplementary Figure 1: Tongue tumor invasion score. Invasion was scored in a blinded fashion by the expert pathologist (S.F.) as follows: 0 = no evidence of invasion, 1 = malignant cells deep to basal cell layer, no invasion into muscle; 2 = minor invasion into striated muscle; 3 = invasion through the full depth of striated muscle. Representative images of H&E stained sections are shown. Magnification × 100.

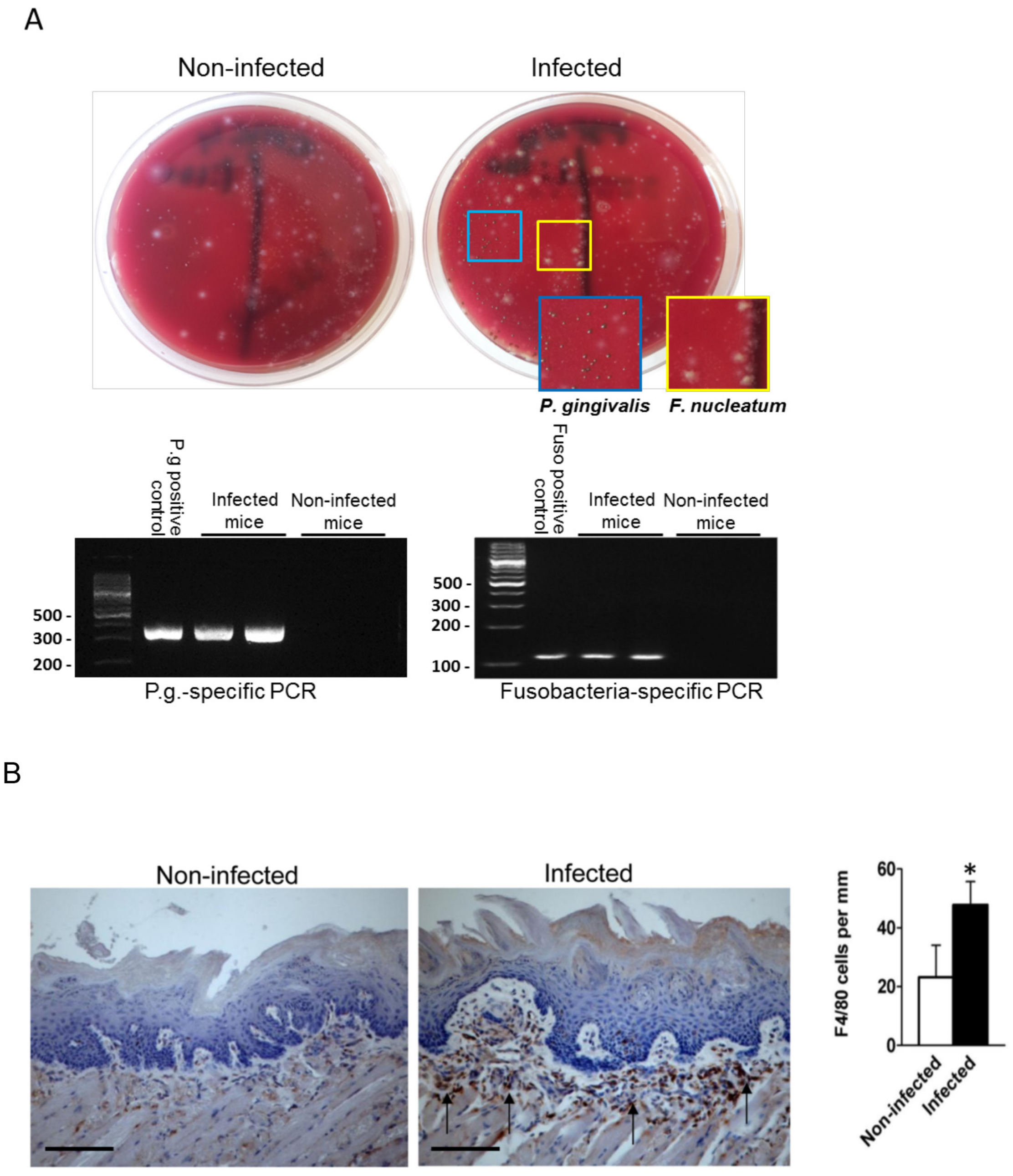

Supplementary Figure 2: A. Recovery of *P. gingivalis* and *F.nucleatum* from tongues of infected mice. The presence of both bacteria on the tongue surface was confirmed by swabbing, culturing and PCR with primers specific for *F. nucleatum* and *P. gingivalis*, as described in Methods. Top: representative images of bacterial colonies recovered from non-infected (left) and infected (right) mice are present. Insets: enlarged images of the areas delineated by blue line (representing *P. gingivalis* colonies, identified by black pigmentation) and by yellow line (representing *F. nucleatum* colonies, identified by yellow-gray color) are shown. Bottom: identity of *P. gingivalis* (left) and *F.nucleatum* (right), confirmed by colony PCR. **B**. Increased macrophage infiltration in tongue sub-epithelium of mice infected with *P. gingivalis/F. nucleatum*. Left and Middle: Immunostaining with anti F4/80 (black arrows) of tongue specimens harvested on experimental week 18 from non-infected (left) and infected (middle) mice. Original magnification × 200. Right: Quantification of F4/80-positive macrophages. Data shown are mean ± SD, $n = 5$ mice per condition. $^{**}P < 0.01$.

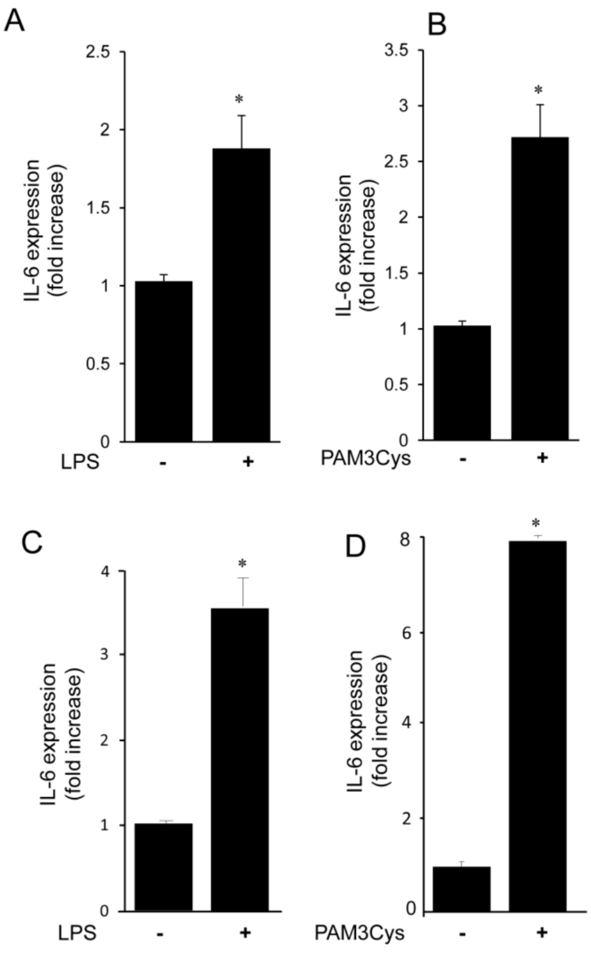

Supplementary Figure 3: Increase in IL-6 expression by SCC cell lines in response to treatment with TLR 4 and TLR 2/1- specific ligands. SCC-25 **A, B.** and CAL 27 **C, D.** cells were remained untreated or treated for 4 h with either LPS (A, C) or PAM3Cys (B, D). IL-6 expression was assessed by qRT-PCR. *$P < 0.05$.

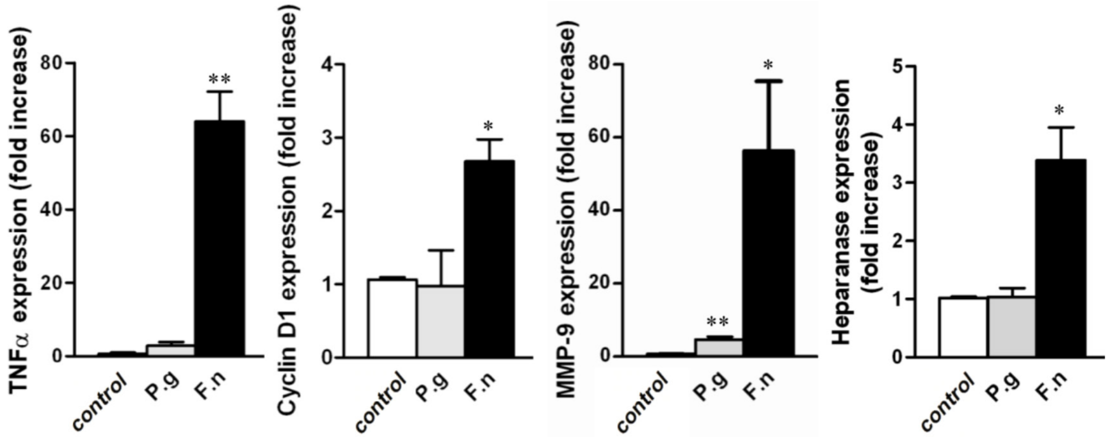

Supplementary Figure 4: Increased expression of SCC-promoting biomolecules by SCC-25 cells following co-culture with either P. *gingivalis* or F. *nucleatum*. SCC-25 cells were cultured alone or in the presence of P. *gingivalis* or F. *nucleatum*. qRT-PCR was used to assess expression of TNFa (4 hours of co-culture), cyclin D1, MMP9 and heparanase (24 h of co-culturing). *$P < 0.05$. **$P < 0.01$.
